# Rediscovering Medicinal Amazonian Aromatic Plants:* Piper carpunya* (Piperaceae) Essential Oil as Paradigmatic Study

**DOI:** 10.1155/2019/6194640

**Published:** 2019-01-01

**Authors:** José L. Ballesteros, Massimo Tacchini, Antonella Spagnoletti, Alessandro Grandini, Guglielmo Paganetto, Luca Maria Neri, Arianna Marengo, Letizia Angiolella, Alessandra Guerrini, Gianni Sacchetti

**Affiliations:** ^1^Department of Life Sciences and Biotechnology, Pharmaceutical Biology Lab., Technopole Lab. Terra&Acqua Tech (Research Unit 7), University of Ferrara, P.le Chiappini 3, 44123 Ferrara, Italy; ^2^Department of Life Sciences, Politécnica Salesiana University, 17025 Quito, Ecuador; ^3^Department of Morphology, Surgery and Experimental Medicine, Section of Human Anatomy, University of Ferrara, Via Fossato di Mortara 66, 44121 Ferrara, Italy; ^4^Department of Drug Science and Technology, University of Torino, Via P. Giuria 9, 10125 Torino, Italy; ^5^Department of Public Health and Infectious Diseases, Sapienza University of Rome, P.le Aldo Moro 5, 00165 Rome, Italy

## Abstract

*Piper carpunya *Ruiz & Pav. (Piperaceae) is a perennial aromatic shrub of Amazonian area of Ecuador and Peru, belonging to the ethnomedicine of these countries. The traditional preparations of the crude drug (fresh leaves used topically as is, and dried leaves in infusions or decoctions) are known for anti-inflammatory, antiulcer, antidiarrheal, antiparasitic effects, and wound healing properties. In light of this traditional evidence, chemical composition (GC-MS) and biological activity, i.e., antioxidant, antifungal (yeast) capacities, and genotoxicity, of Amazonian* P. carpunya* leaf essential oil (EO) have been investigated in order to valorize some of the putative ethnomedical effects. The EO was obtained through steam distillation of fresh leaves (yield: 7.6 g/kg [0.76%]; refractive index at 20°C: 1.49; density: 0.928 g/mL). Chemical characterization performed through GC-MS evidenced the presence of 21 compounds (96.2% of the total). The most abundant constituents were piperitone (26.2%), limonene (9.5%), elemicin (7.2%), and *β*-phellandrene (5.6%).* In vitro* DPPH antioxidant assay showed a weak radical scavenging ability (IC_50_) if compared to positive control. Low bioactivity of the EO was also demonstrated against yeast, but it showed an interesting synergistic activity (FIC index of EO+fluconazole) against* Candida *sp. strains. Ames test evidenced the safety of the EO concerning genotoxicity.

## 1. Introduction

This study is part of a large research project characterized by the biodiversity mapping of Amazonian Ecuador flora belonging to ethnomedical traditional uses through chemical and biological characterization of EOs [1, 2].

Since most of the plant species used as traditional health remedies are characterized by flavours and aromatic smell, the study of EOs is one of the best strategies to draw a research profile matching biodiversity and phytomedicine.* Piper carpunya* Ruiz & Pav (syn.* Piper lenticellosum* C.D.C.) is a perennial shrub belonging to the Piperaceae botanical family, widely distributed in the Amazonian area of Colombia, Ecuador and Peru. Traditionally, the aromatic* Piper* species are used in Latin America as analgesics in pain management, toothache and wound treatments [3].* P. carpunya* has instead more specific and slightly different ethnomedical applications. In fact, fresh and dried leaves of* P. carpunya*, mainly known with the vernacular name of “*guaviduca,*” are widely used in traditional preparations (i.e., decoctions or direct application) in tropical and subtropical South American Countries to treat generic inflammations (i.e., bronchitis and fever), topical infections (i.e., vaginal candidiasis), gastrointestinal disorders (i.e., ulcers, diarrhea and parasitic intestinal infections) and generic digestive problems [4, 5].

Not many papers were published about* P. carpunya*. Some of them deal with the above mentioned uses: for example, the capacity of traditional preparations of* P. carpunya* to protect* in vivo *against gastric disorders induced by gastric harmful drugs or* Helicobacter pylori* infections [4]. Other papers, instead, reported the phytochemical composition of the EO obtained by the leaves of* P. carpunya*, mainly characterized by terpenes [6–8].

Despite the abundant scientific reports about* Piper* species, the knowledge about* P. carpunya* EO is still scarce. Therefore, the aim of the present paper is to characterize the EO obtained from the leaves of the Ecuadorian* P. carpunya* and to evaluate its biological activities. A phytochemical fingerprint was performed through gas chromatography coupled with mass spectrometry (GC–MS) in order to chemically characterize the EO. It was subsequently tested* in vitro *for antioxidant and antimicrobial evaluations, also through synergistic and time-killing assays, in order to verify the traditional uses assuming that the bioactivities were mainly due to the terpene fraction. For this reason, the most abundant terpene compounds detected in the EO were also tested for the same bioactivities, trying to identify those molecules putatively responsible of the biological properties. Finally, in order to assess the safety of the* P. carpunya* EO, Ames test with* S. typhimurium* strains was performed to check possible genotoxic implications.

## 2. Materials and Methods

### 2.1. Plant Material and Essential Oil

*Piper carpunya *Ruiz & Pav leaves of adult plants from three different stocks were collected in November 2014 at balsamic period (before flowering) from wild shrubs growing in the outskirts of the Kutuku Experimental Station, province of Morona Santiago, Ecuador. After harvesting, the* P. carpunya* leaves were* in situ *immediately subjected to hydrodistillation using a portable Clevenger-type apparatus. The EO was extracted by steam distillation (3 h) of* P. carpunya* fresh leaves (8 kg) using deionized water (8 L). The refractive index was measured at 20°C using the refractometer Abbe 60, while density was checked using a densitometer DCE-DB 600. The essential oil samples were then stored in amber airtight glass vials at 18±0.5°C to prevent degradations prior to analyses. A specimen of* P. carpunya* EO was deposited at Salesian Polytechnic University (Quito, Ecuador) (voucher specimen: HUPS-pi-002). Aliquots of the EO samples were then transferred maintaining the storage conditions of the original specimen to the Department of Life Sciences and Biotechnology, University of Ferrara (Ferrara, Italy) for chemical and biological analyses.

### 2.2. Chemicals

All solvents and chemicals employed for chemical and biological analyses were chromatographic grade. Solvents, pure compounds, employed as positive reference, were all purchased from Sigma–Aldrich Italy (Milano, Italy; www.sigmaaldrich.com).

### 2.3. Gas Chromatographic Coupled with Mass Spectrometry (GC-MS) and Flame Ionization Detection (GC-FID) Analyses

*P. carpunya* EO was analyzed by a Varian GC- 3800 gas chromatograph equipped with a Varian MS-4000 mass spectrometer using electron impact and hooked to NIST library. A Varian FactorFour VF-5ms poly-5% phenyl-95%-dimethylsiloxane column (internal diameter, 0.25 mm; length, 30 m; film thickness, 0.15 *μ*m) was used. The following conditions were adopted: injector temperature 250°C, carrier (Helium) flow rate 1 mL/min and split ratio 1:50. Oven temperature was as follows: from 55 to 100°C at a rate of 1°C/min, from 100 to 250°C at a rate of 5°C/min and then kept constant at 250°C for 15 min. One microliter of sample dissolved in methanol was injected. For the calculation of the relative retention time (RI), a mixture of C8 to C32 hydrocarbons (Sigma-Aldrich), was previously injected into the system applying the same method used to analyze EO. The constituents of the volatile oil were identified by comparing their GC relative retention times, RI and the MS fragmentation pattern with those of other EOs of known composition, with pure compounds and by matching the MS fragmentation patterns, as well as retention indices, with the above mentioned mass spectra libraries and with those in the literature [12, 13]. The MS conditions were ionization voltage, 70 eV; emission current, 10 *μ*Amp; scan rate, 1 scan/s; mass range, 29–400 Da; trap temperature, 150°C and transfer line temperature, 300°C. For the quantitative analysis a ThermoQuest GC-Trace gas-chromatograph equipped with a FID detector and the same column above described were used. The operating conditions for gas chromatograph were reported above. FID temperature was 250°C. The oil percentage composition was performed by the normalization method from the GC peak areas, without using correction factors.

### 2.4. Biological Activities

Antioxidant properties and antimicrobial activity against different strains of human pathogen yeast* Candida albicans *were performed using the EO and the most abundant compounds, as putative most responsible agent of biological properties. With specific reference to antimicrobial activities, synergistic interactions were also checked in order to explore the possibility of increasing the biological efficacy employing a formulation with different ratio of EO and fluconazole as synthetic drug commonly used in pharmaceuticals for treating candidiasis. Ames test was finally performed to assess safety of* P. carpunya* EO for what concerns genotoxicity. All the bioactivities were performed using proper negative and positive controls [14]. All the data reported are the average of three determinations of three independent experiments.

#### 2.4.1. DPPH Antioxidant Activity: High-Throughput RDSC Assay

A DPPH (2,2-diphenyl-1-picrylhydrazyl) stock solution (0.208 mM in 50% ethanol) was prepared and kept at 4°C in the dark, according to the method reported for High-Throughput RDSC assay [15]. Ethanol stock solutions of Trolox® (50%) (160 *μ*M),* P. carpunya* EO (2 mg/mL), piperitone, limonene, 1,8-cineole, methyleugenol and thymol (20 mg/mL) were also prepared and stored at 4°C. Starting from the stock solutions, 96-well microplates were filled with 200 *μ*L/well of the following samples progressively less concentrated (dilution ratio 1:2): (i)* P. carpunya* EO + DPPH ethanol solution 50% (100 *μ*L + 100 *μ*L); (ii) Piperitone (limonene, 1,8-cineole, methyleugenol) + DPPH ethanol solution 50% (100 *μ*L + 100 *μ*L); (iii) Trolox® (or thymol) + DPPH ethanol solution 50% (100 *μ*L + 100 *μ*L; as positive control); (iv) DPPH ethanol solution 50% + ethanol 50% (100 *μ*L + 100 *μ*L; as negative control); (v) ethanol solution 50% (200 *μ*L; as blank). The concentration ranges were as follows: 7.8-1000 *μ*g/ml for* P. carpunya* EO, thymol, limonene, methyleugenol and 1,8-cineole; 78-10000 *μ*g/ml for piperitone; 0.6-80.0 *μ*M for Trolox and each concentration was tested in triplicate. 0.104 *μ*M was the DPPH concentration in all wells. The 96-well microplates were then incubated in agitation (100 rpm) for 40 min at room temperature in the dark. After incubation, the 96-microwells plates were checked with microplate reader (Microplate Reader 680 XR, Biorad) at 515 nm. Antioxidant activities were expressed as IC_50_ (concentration providing DPPH 50% inhibition) and calculated from inhibition curves obtained by plotting inhibition percentage against EO and pure compounds concentration and as *μ*mol/g of Trolox equivalent [16]. All experiments were assessed in triplicate and values were reported as mean ± SD (standard deviation).

#### 2.4.2. Antimicrobial Activity against Candida spp.: Microbroth Dilution Method

Antimicrobial tests were carried out by the microbroth dilution method using three* Candida *spp. strains:* Candida albicans *(AIDS6; fluconazole resistant clinical strain isolated from a HIV patient),* Candida glabrata *(FLU 43976; resistant to fluconazole), and* Candida albicans *(ATCC 24433; from American Type Culture Collections; sensitive to fluconazole). The Minimum Inhibitory Concentration (MIC) was determined through the microdilution method in accordance with “Clinical and Laboratory Standards Institute/National Committee for Clinical Laboratory Standards” (CLSI / NCCLS).* P. carpunya* EO and fluconazole, used as positive control, were diluted on 96-well microplate, obtaining 8 different concentrations (from 48.8 to 6250 *μ*g/mL and from 0.5 to 64 *μ*g/mL respectively), tested in triplicate. In each microwell,* Candida* strains were inoculated to reach a final concentration of 2.5x10^3^ CFU/mL and incubated for 48 hours at 30°C with the EO samples and fluconazole at different concentrations; the final total volume in each well was 200*μ*L [17]. The* Candida* culture media were RPMI-1640 supplemented with 3-(N-morpholino) propanesulfonic acid (MOPS) at pH 7.0; Tween 20 (0.2%; Sigma-Aldrich, St. Louis, MO, USA) was added to improve the solubility. The MIC was determined by direct observation of the turbidity of the culture medium. The MIC values were represented by the lowest concentration at which no turbidity of the medium was observed. Then, the Minimum Fungicidal Concentration (MFC) was checked by transferring 10 *μ*L of the culture medium of each well into a new well with Saboraud Dextrose Agar (SDA) and incubating at 30°C for 48 hours. The final volume in each well was 200 *μ*L. The MFC was considered as the lowest concentration that did not determine* Candida* growth. Culture media and Tween 20 (0.2%) solution (200*μ*L) were used as negative control.

#### 2.4.3. Synergy Test

Synergistic evidence of anti-*Candida* activity was checked through checkerboard test using EO and fluconazole [18] at the same experimental conditions employed for MIC and MFC determination. Eight serial dilutions (1:2) of* P. carpunya* and fluconazole were used to build an 8x8 square matrix that allowed to assay 64 different combinations of EO and drug concentration in one 96-well microplate. The X-axis of the matrix corresponded to the EO concentration gradient dilutions (range: 48.8 - 6250 *μ*g/mL); the Y-axis, instead, was represented by the fluconazole gradient dilutions (range: 0.5 - 64 *μ*g/mL). The synergistic activity was expressed as fractional inhibitory concentration (FIC_index_) and was calculated as follows:

(1) $$\begin{array}{c} {FIC}_{index}={FIC}_{EO}+{FIC}_{fluconazole} \end{array}$$

where FIC_EO_ = MIC_EO+fluconazole_/MIC_EO_; FIC_fluconazole_ = MIC_fluconazole+EO_/MIC_fluconazole_.

The same procedures to detect FIC_index_ for MIC values were performed to check possible synergistic effect for MFC.

Legend: FIC_index_values ≤ 0.5 means the presence of synergistic effect; 0.5<values≤2 means additive or indifferent effect; values > 2 means antagonistic effect [18].

#### 2.4.4. Time Killing

To confirm the synergistic activity, a time-killing fungicide curve was performed [9, 19]. Based on the synergistic results,* C. albicans* AIDS6 strain was used to test* P. carpunya* EO and fluconazole combination. AIDS6 strain was grown in tubes filled with Sabouraud dextrose broth (SDB, LTD Oxoid, Basingstoke, Ampshire, England) for 24 h at 28°C. Then, AIDS6 cultures were centrifuged, washed and resuspended to reach a concentration of 2.5 × 10^5^ CFU/mL in RPMI-1640 buffered with MOPS at pH 7.0 and incubated at 28°C. The following four samples were tested in triplicate: (i)* P.carpunya* EO (MIC value); (ii) fluconazole (MIC value); (iii)* P. carpunya *EO and fluconazole in combination (best FIC_index_ concentrations); (iv)* P. carpunya *EO and fluconazole in combination (the first value higher than the best FIC_index_ concentrations). Tween 20 (0.2%) has been added to increase the solubility of the EO. The same procedure was used to prepare the negative controls (AIDS6 culture and Tween 20, 0.2%). Each sample was incubated at 30°C and gently shacked (100 rpm). Cell growth was monitored at time 0, after 1h, 2h, 4h, 6h and 24h; 100 *μ*L of the AIDS6 culture at each monitoring time was serially diluted (dilution range 1-10^5^). One hundred microlitres (100*μ*L) of each dilution was seeded in Petri dishes (90 mm diameter) containing SDA and incubated for 48 h at 30°C in the dark. After incubation, the grown AIDS6 colonies were counted [10].

#### 2.4.5. Genotoxicity Assay (Ames Test)

To investigate the possible genotoxic activity of* P. carpunya* EO, Ames test employing the* Salmonella typhimurium* strains TA97a, TA98, TA100 and TA1535 was performed [11, 20]. An inoculum of each bacterial strain culture (100*μ*L) was added to 20 ml of Nutrient Broth and incubated at 37°C in an orbital shaker (120 rpm) until a microbial concentration of approximately 2 x 10^8^ bacteria/ml is reached. Petri dishes (90 mm diameter) containing a basic agar and low concentrations of histidine and biotin were topped with 2 ml of molten agar (45°C) prepared with 100 *μ*L of fresh bacterial culture and 100 *μ*L of EO diluted in DMSO (dilution range: 0.1-10 mg/plate). The mutagenic activity was determined on the basis of the counted colonies of each* Salmonella* strain in plates treated with different concentrations of* P. carpunya* EO with metabolic activation [addition of 500 *μ*L of S9 mix dissolved in a solution of KCl (33mM), NADPH (4mM), MgCl_2_ (8mM), and glucose-6-phosphate (5mM)] or, alternatively, without metabolic activation (500 *μ*L of 7.4 pH phosphate buffer). Positive controls were set up with 2 *μ*g/plate of 2-aminoanthracene for all* Salmonella* strains cultured with metabolic activation; and 2 *μ*g/plate of 2-nitrofluorene for strains TA97a, TA98, TA1535, and 1 *μ*g/plate of sodium azide for TA100 strain, for Ames test performed without activation by S9 mix. Negative controls were set up with 100 *μ*L of DMSO, both with and without S9 mix. The plates were incubated for three days at 37°C and colonies were counted (Colony Counter 560 Suntex, Antibioticos, Italy). All the mutagenic assays were performed in triplicate and the results, integrated by statistical analyses, were considered positive when the number of colonies of revertants was at least double if compared to those of the negative controls [11, 20].

### 2.5. Statistical Analyses

All the experiments were performed in triplicate. IC_50_values were assessed by logarithmic regression curves with 95% confident limits. Relative standard deviations and statistical significance (*Student's T *test; p±0.05) were calculated using software STATISTICA 6.0 (StatSoft Italia srl, Vigonza, Italy).

## 3. Results

### 3.1. Chemical Fingerprinting of P. carpunya Essential Oil

**Figure 1:**
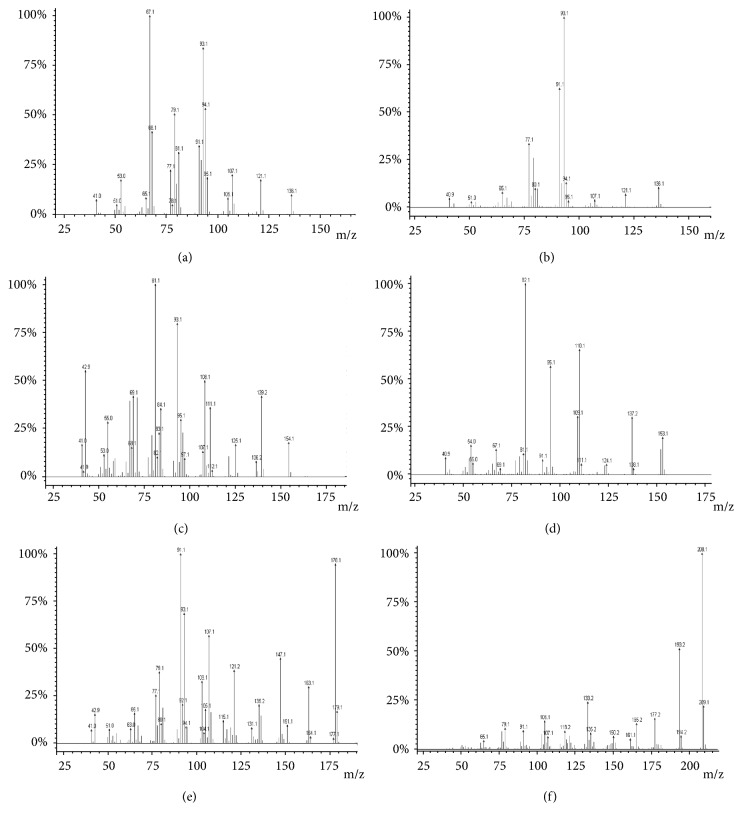
Experimental mass-spectra of (a) limonene, (b) *β*-phellandrene, (c) 1,8-cineole, (d) piperitone, (e) methyeugenol and (f) elemicin.

Fresh leaves of* P. carpunya* were collected during the balsamic period (before flowering) and directly steam-distilled to obtain the essential oil. The distillation yield was of 0.76% (w/w%), the refractive index of 1.49 ± 0.02, and the density of 0.928 ± 0.040 g/mL. The colour was pale yellow, and the smell was persistent and strongly aromatic, similar to that of pepper. GC-MS analyses were performed to determine the chemical characterization of the essential oil: fortytwo (42) compounds (96.2% of the total) were identified. Piperitone (26.2%), limonene (9.5%), elemicin (7.2%), *β*-phellandrene (5.6%), methyleugenol (4.5%), and 1,8-cineole (4.0%) were the most abundant (Table 1): their experimental mass-spectra were reported in Figure 1.

### 3.2. Antioxidant Activity of P. carpunya Essential Oil: DPPH Assay

The DPPH assay was performed to test the antioxidant capacity of* P. carpunya* EO and of the main compounds detected in the phytocomplex. The results were compared to those achieved with positive control Trolox® and thymol, taken as reference terpene with well-known antioxidant activity (Table 2) [21].The antioxidant activity of* P. carpunya* EO was moderately interesting with values very far from both pure compound thymol and Trolox®. Piperitone, limonene, 1,8-cineole, and methyleugenol were tested alone as pure compounds: only the last was active, but the activity of EO could not be explained by these molecules. Their mixture or other minor compounds could be responsible of the DPPH capacity.

### 3.3. Antimicrobial Activity against Candida sp.: Minimum Inhibitory Concentration

The possible synergistic effects of EO and fluconazole (the first-line agent in the candidiasis treatment) were tested at different doses to evaluate different mutual relations. The fractional inhibitory concentration index (FIC_index_) was used as synergy evaluation parameter and was considered synergistic at values less or equal to 0.5 [18].  Table 3 summarizes the results obtained, the activity of the antifungal fluconazole, and the EO tested individually and in combination. The MIC and MFC values for* P. carpunya* EO were found to be 781 *μ*g/mL and 1562 *μ*g/mL, respectively, for* C. albicans *(ATCC 24433); the same results (MIC: 1562 *μ*g/mL; MFC: 1562 *μ*g/mL) were checked for* C. albicans *(AIDS6) and* C. glabrata *(FLU 43976).* C. glabrata *(FLU 43976) and* C. albicans *(ATCC 24433) showed the same sensitivity if treated with fluconazole (MIC: 32 *μ*g/mL; MFC: 32 *μ*g/mL).* C. albicans *(AIDS6) showed instead that it is more resistant (MIC: 128 *μ*g/mL; MFC: 128 *μ*g/mL).

### 3.4. Sinergy and Time Killing 

The presence of synergistic effects of a mixture characterized by different concentrations of* P. carpunya* EO and fluconazole (1:1) was checked on the three* Candida *sp.strains. The MIC and MFC results that gave the best FIC_index_ data are reported in Table 3. The MIC and MFC values obtained with EO plus fluconazole solution (EO: 391 *μ*g/mL; fluconazole: 4 *μ*g/mL) were 4-folds lower than those displayed by the sole EO for both* C. albicans *(AIDS6) and* C. glabrata *(FLU 43976). The MIC and MFC obtained with the sole fluconazole were 32-folds higher than those achieved with the mixture of EO and synthetic drug for* C. albicans *(AIDS6), while they were 8-folds for* C. glabrata *(FLU 43976). No synergistic effects were evidenced for* C. albicans *(ATCC 24433).

**Figure 2:**
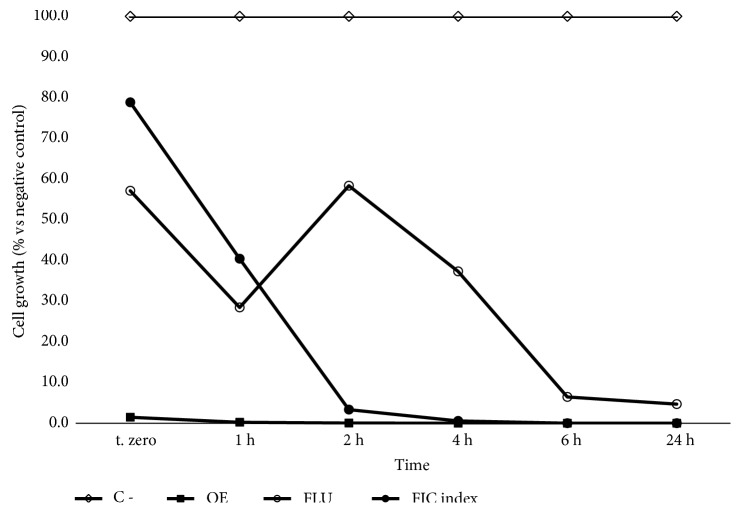
Time-killing curves of MICs (Minimum Inhibitory Concentrations, *μ*g/mL) of* P. carpunya *(EO), fluconazole (FLU) and their combination (FIC index) against* C. albicans *(AIDS6).

The synergistic effect of* P. carpunya* EO with fluconazole was confirmed by time-kill curve experiments performed employing the* C. albicans *(AIDS6) strain since it showed the best FIC_index_. The strain (cell density of 1–5 x 10^5^ CFU/mL) was exposed to MIC values of* P. carpunya* EO, of fluconazole alone and of the EO-fluconazole mixture that displayed the best FIC_index_ (Figure 2). Time-kill analysis showed that the mixture of* P. carpunya *EO and fluconazole almost totally inhibited the growth of the* Candida* strain over 24 hours compared to the negative control. In particular, after only 2 hours of exposure, the microbial growth was almost completely reduced to values similar to those detected after 24 h.

### 3.5. Ames Test: Genotoxic Activity

In order to assess the genotoxic effect of* P. carpunya *EO, induction or suppression of revertant colonies was examined in* Salmonella typhimurium* strains. The number of spontaneous revertants of the four strains with or without S9 metabolic activation was determined in each set of experiment and indicated as untreated sample in Table 4.

Different concentrations of the* P. carpunya *EO did not show any genotoxic effect on* Salmonella *TA97a, TA98, TA100 and TA1535 strains. Just two doses exhibited mutagenic activity (t/c ≥ 2) against TA1535 and TA97a strain, without metabolic activator (S9 mix) at 500 and 1000 *μ*L/plate, respectively. On the other hand, in presence of S9 mix microsomal fraction, the only TA1535 strain evidenced a mutagen sensitivity in presence of the EO both at 500 and 1000 *μ*L/plate. In any case, no dose-response trend was observed suggesting the conclusion of the safety of* P. carpunya* EO with regard to genotoxicity.

## 4. Discussion

For the past several years our research group has been involved in studying Amazonian medicinal plants through their chemical and biological fingerprinting starting from ethnomedical information. These research projects contributed to expanding the knowledge about those plants which are rarely or never studied but which may potentially play a role in improving the efficacy and safety of pharmaceuticals and health products. Moreover, Amazonian plants are particularly interesting since the Amazonian basin is one of the most important biodiversity hotspots, where the ecological conditions and the high density of species per unit area drive the plant secondary metabolism to biosynthetic pathways which are particularly rich in different chemical structures [22, 23]. Therefore, the present research is part of a large project aimed to characterize the Amazonian medicinal flora, with particular reference to aromatic plants which represent the largest part of the sources used in traditional medicinal preparations. Since most of the plant species used as traditional health remedies are characterized by flavour and aromatic smell, the study of EOs is one of the best strategies to draw a research profile matching biodiversity and phytomedicine. For this reason, the chemical fingerprinting is focused on the characterization of EOs, while the biological activities are assayed through* in vitro* tests to confirm traditional properties or to find new health applications [1, 2]. Starting from these premises, the purpose of the current research was to study the chemical characterization and biological activities of Amazonian* P. carpunya* EO.

### 4.1. Chemical Characterization of P. carpunya Essential Oil

As far as we know, only few papers dealt with the chemical composition of* P. carpunya* EO. However, comparing our results with those reported for* P. carpunya* EO from Peru emerged important differences [7]. From a qualitative point of view, every compound detected in the* P. carpunya *EO from Peru [7] was present in our EO from Amazonian Ecuador, but in smaller quantities. The latter obtained by Ecuadorian plants showed the main abundance of piperitone (26.2%), limonene (9.5%), elemicin (7.2%), *β*-phellandrene (5.6%), methyleugenol (4.5), and 1,8-cineole (4.0%), while the analogous EO obtained by Peruvian plants was reported to contain safrole, 1,8-cineole, *α*-terpinene, p-cymene, spathulenol and bycyclogermacrene as the terpene compounds occurring in highest amount. These results highlight the high plasticity of the* P. carpunya *species in adapting to different environmental and allelopathic conditions, such as those of Peru and Amazonian Ecuador, and its capacity to produce an EO with a strongly different composition.

### 4.2. Antioxidant Activity of P. carpunya Essential Oil (DPPH Assay)

The oxidative stress is generally considered the starting point for the onset of several diseases (e.g., tissues acute and chronic inflammation) usually occurring through several complex mechanisms. Because the EO of* P. carpunya* is traditionally known to be used as treatment of generic inflammations (e.g., bronchitis, fever) [5] a preliminary antioxidant evaluation has been performed through DPPH assay. The promising results were those expressed by* P. carpunya* EO, suggesting both the possible efficacy of the traditional use of the leaves of* P. carpunya* as anti-inflammatory agent and the use of the Amazonian EO in modern preparations (e.g., antiageing products). However, the lower IC_50_ value detected for* P. carpunya* EO than that of the sole piperitone (and other tested pure compounds), which means a higher bioactivity of the EO than that of the pure compound, might indicate a possible noteworthy bioactivity of the less abundant, or the presence of synergistic interactions among molecules, as often demonstrated for EO [24–26].

### 4.3. Synergistic Antimicrobial Activity against Candida sp. and Time-Killing Evidences

Many plant-derived preparations used in traditional ethnomedicines have been recorded in different pharmacopoeias as agents used to treat various infections and more often as source of effective drugs [15, 27, 28]. In general, EOs are largely known as effective antimicrobial agents [13, 29], but in our study the most interesting evidence is given by the synergistic activity emerged by the combination of* P. carpunya *EO and fluconazole as synthetic drug. In fact, the possibility to enhance bioactivity of health products combining natural products, such as EOs, and synthetic drugs through synergistic interactions gives the opportunity to reduce the amount of synthetic active compounds, reducing at the same time their adverse effects without limiting the therapeutic efficacy. This kind of results obviously highly valorizes the EO of* P. carpunya* from Amazonian Ecuador as possible ingredient in modern anti-candidiasis pharmaceuticals, even more if we consider the activity of the synergistic mixture against resistant fluconazole clinical isolated strains and time-killing curve that show the dramatic reduction of microbial growth after just 2 h.

### 4.4. Genotoxic Safety of P. carpunya Essential Oil

To complement the awareness on the efficacy and the safe possible use of* P. carpunya* EO, Ames test was carried out [17, 20]. EOs can be used topically or orally administered and the careful examination of possible mutagenic properties is required to confirm and assure the safety of their use [17]. Moreover, some evidence of possible genotoxic activities exerted by EOs is emerging [17]. Traditional uses are by no means warrants of safety since complex set of symptoms and cause-effect relationships are not easily recognized and identified by the population [17, and references therein]. Our results pointed out the safety of the* P. carpunya* EO with regard to genotoxicity, suggesting also by this aspect the potentiality of its health uses.

## 5. Conclusions

The present work provides for the first time phytochemical and biological evidence about* P. carpunya* EO obtained from fresh leaves collected from plants in the Amazonian Ecuador. The chemical characterization of the EO showed important differences in comparison with other EOs from the same species but from different geographical areas: these results reflect the adapting behaviour of plants to different environments.

The* in vitro* biological activities suggested, in general, the correct traditional use of the aromatic leaves and their preparations (e.g., topical infections remedy, such as vaginal candidiasis), and their safety under a genotoxic point of view. Particularly promising resulted synergistic activity emerged with aliquots of* P. carpunya *EO and fluconazole, suggesting interesting opportunities to employ the Amazonian Ecuador EO as ingredient in anti-candidiasis products in which the reduced amount of synthetic drug could lead to the reduction of the possible adverse effects without compromising therapeutic efficacy.

## Figures and Tables

**Table 1:** Chemical characterization of *P. carpunya *EO performed by GC-MS and GC-FID analyses.

**N**°	**Compound** ^**a**^	**ID Method** ^**b**^	**RI calc** ^**c**^	**RI lit** ^**d**^	%^**e**^
1	*α*-thujene	RI, MS	920	924	0.2
2	*α*-pinene	Std	927	932	3.4
3	sabinene	Std	965	969	1.4
4	*β*-pinene	Std	970	974	1.1
5	myrcene	RI, MS	985	988	1.4
6	*δ*-2-carene	RI, MS	995	1001	0.2
7	*α*-phellandrene	RI, MS	1003	1002	1.1
8	*δ*-3-carene	RI, MS	1004	1008	0.3
9	*α*-terpinene	RI, MS	1012	1014	3.9
10	p-cymene	RI, MS	1019	1020	3.4
11	limonene	Std	1023	1024	9.5
12	*β*-phellandrene	RI, MS	1024	1025	5.6
13	1,8-cineole	Std	1026	1026	4.0
14	*trans*-E-ocimene	RI, MS	1041	1044	0.2
15	*γ*-terpinene	RI, MS	1051	1054	3.0
16	*cis*-sabinenehydrate	RI, MS	1065	1060	0.2
17	linalool	Std	1099	1095	1.5
18	1,3,8-p-menthatriene	RI, MS	1109	1110	0.3
19	3-thujen-2-ol	RI, MS	1168	1167	2.4
20	terpinen-4-ol	Std	1174	1174	0.3
21	neoisodihydrocarveol	RI, MS	1233	1229	0.6
22	cuminaldehyde	RI, MS	1237	1238	0.3
23	piperitone	Std	1250	1249	26.2
24	*α*-terpinen-7-al	RI, MS	1279	1283	1.3
25	safrole	RI, MS	1283	1285	2.2
26	thymol	Std	1293	1289	0.9
27	carvacrol	RI, MS	1300	1298	0.7
28	*α*-terpinylacetate	RI, MS	1354	1343	1.3
29	eugenol	Std	1361	1356	0.9
30	*α*-copaene	RI, MS	1374	1374	0.4
31	*β*-elemene	RI, MS	1387	1389	0.2
32	*β* -longipinene	RI, MS	1397	1401	0.2
33	methyleugenol	RI, MS	1401	1403	4.5
34	*β*-caryophyllene	Std	1408	1417	0.3
35	p-cymen-7-ol acetate	RI, MS	1422	1421	1.5
36	germacrene D	RI, MS	1474	1485	1.4
37	bicyclogermacrene	RI, MS	1488	1500	1.2
38	elemicin	RI, MS	1551	1555	7.2
39	*trans*-nerolidol	Std	1561	1561	0.2
40	spathulenol	RI, MS	1574	1577	0.7
41	globulol	RI, MS	1583	1585	0.5
42	viridiflorol	RI, MS	1592	1592	0.1

Total					96.2
Hydrocarbon monoterpenes					35.0
Oxygenated monoterpenes					41.2
Hydrocarbon sesquiterpenes					3.7
Oxygenated sesquiterpenes					1.5
Phenylpropanoids					14.8

^a^ Compounds are listed in order of elution from a Varian VF-5ms column.

^b^ Identification method adopted for each compound.

^c^ Retention indices calculated on a Varian VF-5ms column.

^d^ Relative area percentage (peak area relative to total peak area %).

**Table 2:** Antioxidant properties of *P. carpunya* EO evaluated by DPPH assay. The values, expressed as IC_50_, were compared to thymol, piperitone, limonene, 1,8-cineole, methyleugenol and Trolox®.

	**DPPH**	
**Samples**	**I** **C**_50_^1^ ** (** ***μ*** **g/mL)**	** TEAC (mmol TE/g)**
*P. carpunya* EO	159.80 ± 3.40	71.88 ± 1.53
piperitone	2278.00 ± 31.74	2.61 ± 0.32
limonene	>1000	<0.89
1,8-cineole	>1000	<2.05
methyleugenol	602.00±5.59	17.87±0.56
thymol	70.80 ± 1.51	160.02 ± 3.45
trolox	3.97 ± 0.40	--

^1^ IC_50_: concentration corresponding to the 50% of the bioactivity;

^2^ TEAC: Trolox equivalent (TE) antioxidant capacity.

**Table 3:** Synergistic effect of *P. carpunya* EO employing fluconazole as synthetic active drug against *Candida *sp.

	**FICindex MIC Findex MCF**		**EO**		**FLU**	
	(EO+FLU) (EO+FLU)		MIC	MFC	MIC	MFC
***C. albicans *(AIDS6)**	0.281**∗** (391+4)*∗∗*	0.281**∗** (391+4)*∗∗*	1562	1562	128	128
***C. glabrata* (FLU 43976)**	0.375**∗** (391+4)*∗∗*	0.375**∗** (391+4)*∗∗*	1562	1562	32	32
***C. albicans *(ATCC 24433)**	**1.063** (781+2)*∗∗*	**0.016** (1562+0.5)*∗∗*	781	1562	32	32

FICindex: fractional inhibitory concentration index; FIC_index_≤ 0.5 means the presence of synergistic effect; 0.5≤FIC_index_ ≤2 means additive or indifferent effect; FIC_index_> 2 means antagonistic effect[9, 10].

*∗* Values displaying a synergistic effect.

*∗∗* Concentration in *μ*g/mL of EO and FLU, respectively, corresponding to FICindex.

MIC: Minimum Inhibitory Concentration (*μ*g/mL); MFC: Minimum Fungicidal Concentration (*μ*g/mL).

EO: *P. carpunya* EO; FLU: fluconazole.

**Table 4:** Ames test performed to check the genotoxic safety (no. of revertants induced) of *P. carpunya* EO. The results are expressed as *average* of number of colonies per plate, followed by the standard deviation (sd). t/c means the ratio between the number of colonies of *Salmonella *strains grown in presence of EO and those of the negative control (DMSO). If t/c ratio is ≥ 2, following a dose-response trend, the extract can be considered as potential mutagen [11].

**Dose level (** *μ * **L/plate)**	**TA97a**		**TA98**		**TA100**		**TA1535**	
	**Average ± s.d.**	**t/c**	**Average ± s.d.**	**t/c**	**Average ± s.d.**	**t/c**	**Average ± s.d.**	**t/c**
***-S9***								
10	149.0 ± 7.6	1.15	24 ± 4.2	0.79	89 ± 9.9	0.81	4.0 ± 2.8	0.57
50	110.5 ± 10.1	0.85	20 ± 5.7	0.66	87 ± 1.4	0.79	10.5 ± 0.7	1.50
100	149.5 ± 8.5	1.15	25 ± 8.4	0.82	77 ± 12.7	0.70	3.0 ± 1.4	0.43
500	65.0 ± 9.7	0.50	29.5 ± 2.1	0.97	46.0 ± 2.8	0.42	36 ± 3.2	5.14*∗*
1000	546.0 ± 31.1	4.20*∗*	26.0 ± 2.8	0.85	8.5 ± 3.5	0.08	8.0 ± 11.3	1.14
5000	12.5 ± 0.7	0.10	25.5 ± 19.1	0.84	19 ± 24.0	0.17	0.0 ± 0.0	0.00
10000	0.0 ± 0.0	0.00	10.5 ± 6.4	0.34	0.0 ± 0.0	0.00	0.0 ± 0.0	0.00
DMSO (100 *μ*L)(	130.0 ± 14.2	1.00	30.5 ± 0.7	1.00	110 ± 7.1	1.00	7.0 ± 2.8	1.00
2-Nitrofluorene (2 *μ*L)	1554 ± 113.3	12.00	716 ± 75.0	23.48	--	--	47 ± 1.8	6.71
Sodium azide (1 *μ*L)	--	--	--	--	688 ± 72.0	6.25	--	--
***+S9***								
10	67.5 ± 8.4	0.39	44 ± 2.8	1..06	106 ± 14.1	1.15	9.5 ± 0.7	1.00
50	112.5 ± 9.7	0.66	34 ± 4.2	0.82	84 ± 5.7	0.91	12 ± 1.4	1.26
100	112.5 ± 8.1	0.66	43 ± 14.1	1.04	87 ± 26.9	0.95	11 ± 4.2	1.16
500	157.5 ± 12.1	0.92	35 ± 0.0	0.84	76.5 ± 9.2	0.83	38.5 ± 6.8	4.05*∗*
1000	175.5 ± 10.2	1.03	37.5 ± 6.4	0.90	64.5 ± 9.2	0.70	23.5 ± 33.2	2.47*∗*
5000	0.0 ± 0.0	0.00	25 ± 7.1	0.60	22.5 ± 12.0	0.24	0.0 ± 0.0	0.00
10000	0.0 ± 0.0	0.00	14 ± 4.2	0.34	6 ± 5.7	0.07	0.0 ± 0.0	0.00
DMSO, 100 *μ*L	171.0 ± 6.8	1.00	41.5 ± 2.1	1.00	92 ± 2.8	1.00	9.5 ± 0.7	1.00
2-Aminoanthracene (2*μ*L)	896 ± 10.4	5.24	932 ± 98.0	22.46	912 ± 95.0	9.91	168 ± 18.0	17.68

+S9: experiments performed with metabolic activation (S9 mix).

-S9: experiments performed without metabolic activation (S9 mix).

DMSO: dimethylsulfoxide, negative control; 2-aminoanthracene: positive control for experiments with S9; 2-nitrofluorene: positive control for TA97a and TA98 strains for experiments without S9; Sodium azide: positive control for TA100 and TA1535 strains for experiments without S9.

*∗*Values corresponding to mutagen activity.

## Data Availability

The data used to support the findings of this study are included within the article.
